# Hereditary factors in basal cell carcinoma of the skin: a population-based cohort study in twins

**DOI:** 10.1054/bjoc.1999.0908

**Published:** 1999-12-08


various reasons, as directly and straightforwardly outlined in the discussion of the article. Nevertheless, we are somewhat astonished by the reason for the critical comments by Drs Huland and Heinzer, related for the most part to the fact that they only rely their discussion on non-randomized studies including selected population of patients. In fact, we lack references to well controlled studies. An obvious shortcoming of most of the previously published studies within the field of immunotherapy is the conclusion that the response rate was superior to 'historic controls', although these trials were not randomized, and did not use case-matched controls (Philip et al, 1993).

It has to be emphasized that our study investigated the general applicability of the therapy concept in the management of patients with good performance status (WHO 0–2). Even if it is a possibility that the doses delivered to the patients are lower than proposed to be optimal by Huland and Heinzer, it was clear that in our hands many of the patients (note patients with high performance status) required dose-reduction to be able to manage the treatment at all. The toxicity encountered was in accordance with earlier reported, and included a substantial number of patients with grade 3 and 4 toxicity. Thus, the adverse effects seen deteriorated quality of life in a significant manner of the patients treated with immunotherapy. Publication is also underway for the final evaluation of quality of life for the whole study. This gives at least a further support that the doses used were of clinical significance, i.e. the toxicity was substantial. There exist no conclusive studies that clearly demonstrate a dose–response relationship for cytokines in the clinical situation. Most likely, there is a quite different dose–response correlation for cytokines ('bell-shaped') compared to chemotherapeutics. Thus, it is not clear that 'more is better'.

The survival in our study was similar in the two groups of patients, even in respect to long-term survival. Moreover, the survival was quite comparable with survival data reported in other studies of renal cell carcinoma patients treated with IL-2/$\alpha$-IFN (e.g. Facendola et al, 1995). In fact, median survival in each of the treatment arms was better than that seen in Swedish registry studies. This gives further support that the doses delivered to patients were of clinical significance. The survival analysis did not seem to be different regardless of the time frame of the analysis (from the date of primary diagnosis or the start of the treatment or from the time of first signs of metastatic spread).

There was no obvious initial variation in laboratory parameters or metastatic spread of the disease, which further reduces the risk of differences in prognostic factors between the groups. Furthermore, there exist several previously published studies that support our observations (Steineck et al, 1990; Wagstaff et al, 1995; Ljungberg and Henriksson, 1997).

The past decades have without doubt shown an outstanding increase in the knowledge about tumour immunology and biotherapy. In renal cell carcinoma, the relatively high response figures induced by a biotherapy approach have encouraged extensive clinical studies. We would like to stress that we do not deny the beneficial effects of biotherapy seen by several other authors (e.g. Atzpodien et al, 1995), and agree that there might exist subgroup of highly selected patients with renal cell carcinoma that can really benefit from biotherapy. We also agree that other treatment approaches, like inhalation of IL-2, can be promising. Therefore, we are eagerly waiting the first reported experiences from 1989 of local delivery of IL-2 by Huland and co-workers evaluated in a controlled randomized study.

At present, regardless of our study, there is no standard immunotherapy (a conclusion made in our study) that can be recommended since the results obtained do not suggest an obvious therapeutic benefit for the larger patient population suffering from advanced renal cell carcinoma. It is obvious that there is much need for investigation to find the optimal biotherapy schedule, i.e. a significant increase in survival with an acceptable quality of life.

*R Henriksson*
*Department of Oncology, University Hospital, Umeå, Sweden,*
*P Wersäll*
*Radiumhemmet, Karolinska Institute, Stockholm, Sweden*

### REFERENCES

Atzpodien J, Hänninen LE, Kirchner H, Bodenstein H, Pfreundschuh M, Rebmann U, Metzner B, Illiger H-J, Jakse G, Niesel T, Scholz H-J, Wilhelm S, Pielmeier T, Zabrewski G, Blum G, Beier J, Müller G-W, Duensing S, Anton P, Allhoff E, Jonas U and Poliwoda H (1995) Multiinstitutional home-therapy trial of recombinant human interleukin-2 and interferon alfa-2 in progressive metastatic renal cell carcinoma. *J Clin Oncol* **13**: 497–501

Facendola G, Locatelli MC, Pizzocaro G, Piva L, Pegoraro C, Bobbio Pallavicini E, Signaroldi A, Meregalli M, Lombaardi F, Beretta GD, Scanzi F, Labianca R and Luporini G (1995) Subcutaneous administration of interleukin 2 and interferon-alpha-2b in advanced renal cell carcinoma: a confirmatory study. *Br J Cancer* **72**: 1531–1535

Huland E and Huland H (1989) Locla continuous high-dose interleukin-2: a new therapeutic model for the treatment of advanced bladder carcinoma. *Cancer Res* **49**: 5469–5474

Ljungberg B and Henriksson R (1997) Immunotherapy of metastatic renal cell carcinoma. *Curr Opin Urol* **7:** 252–258

Philip T, Negrier S, Lasset C, Coronel B, Bret M, Baly JY, Merrouche Y, Carrie C, Kaemmerlein P, Chauvin F, Favrot M, Oskam R, Tabah I, Clavel M, Moskovtchenko JF and Mercatello A (1993) Patients with metastatic renal carcinoma candidate for immunotherapy with cytokines. Analysis of a single institutional study on 181 patients. *Br J Cancer* **68**: 1036–1042

Steinec G, Strander H, Carbin B-E, Borgström E, Wallin L, Achtnich U, Arvidsson A, Söderlund V, Näslund I, Esposti P-L and Norell SE (1990) Recombinant leukocyte interferon alpha-2a and medroxyprogesterone in advanced renal cell carcinoma. A randomized trial. *Acta Oncol* **29**: 155–162

Wagstaff J, Baars JW, Wobink G-J, Hoekman K, Eerenberg-Belmer AJM and Hack CE (1995) Renal cell carcinoma and interleukin-2. A review. *Eur J Cancer* **31A**: 401–408

### Hereditary factors in basal cell carcinoma of the skin: a population-based cohort study in twins

**Sir**,

Milan et al (*Br J Cancer* 1998 **78**: 1516–1520) reported on a large twin study investigating the contribution of hereditary factors in basal cell carcinoma (BCC) in Finland. This twin study based on 12 941 adult twin pairs with 43 years follow-up data concluded that genetic factors are not necessary to explain the distribution of BCC in twins. These findings are of major importance; however, a number of points need to be addressed in the analysis before these conclusions can be accepted.

© 2000 Cancer Research Campaign 

1. The first limit to inference is the power of the study, which is insufficient to rule out a genetic effect. Although the CE model was reported as fitting the data best, the confidence intervals for A (additive genetic effects) in the AE and ACE models are very large. Both confidence intervals for A and C (shared environment) contains 0 for the ACE model. A genetic effect as large as 60% cannot be ruled out by this study. Accepting C in place of A for discontinuous traits is logistically difficult and would require a sample size of at least 20 000 twin pairs given the prevalence of basal cell carcinomas of around 2% (Neale et al, 1994).
2. The authors do not explore or comment on the significant common environmental influence that has been observed, which could merely be due to the age-dependence of BCCs. The mean age of onset for sporadic BCC is around 65 years of age, whilst familial tumours usually occur at younger age and are often multiple (Kimonis et al, 1997). Failure to account for age may mask the importance of a heritable component in the data.
3. No account was taken of body sites. In genetically susceptible families, it is well recognized that tumours are more often found on the trunk than the face (Kimonis et al, 1997). By combining all body sites in the analysis, it may have masked a site-specific genetic effect.
4. Loss of heterozygosity studies have shown that 60% of BCCs show loss of chromosome 9q, which harbour the patched (PTC) gene (Gailani et al, 1992). Germline mutations in the PTC gene are found in patients with naevoid BCC syndrome, a family cancer syndrome characterized by multiple early onset BCCs and developmental defects (Johnson et al, 1991). A genetic basis for this disease is therefore likely and can only be adequately discounted in much larger studies using designs that take into account the known biological properties of the disease.

*V Bataille[1,2], H Snieder[2], A MacGregor[2] and T Spector[2]*
*[1]Academic Department of Dermatology, St Bartholomew's and Royal London School of Medicine and Dentistry, London E1, UK; [2]Twin Research and Genetic Epidemiology Unit, St Thomas Hospital, London SE1, UK*

### REFERENCES

Gailani MR, Bale SJ, Leffell DJ et al (1992) Developmental defects in Gorlin's syndrome related to putative tumour suppressor gene on chromosome 9. *Cell* **69**: 111–117

Johnson RL, Rothman AL, Xie J et al (1996) Human homolog of patched, a candidate gene for the basal cell nevus syndrome. *Science* **14**: 1668–1671

Kimonis VE, Goldstein AM, Pastakia B et al (1997) Clinical manifestations in 105 persons with the nevoid basal cell carcinoma syndrome. *Am J Med Genet* **31**: 299–308

Neale MC, Eaves LJ and Kendler KS (1994) The power of the classical twin study to resolve variation in threshold traits. *Behavior Genet* **24**: 239–258

### Hereditary factors in basal cell carcinoma of the skin: a population-based cohort study in twins—reply

**Sir,**

We thank Dr Bataille and her colleagues for their interest in our paper on hereditary factors in basal cell carcinoma of the skin (BCC), based on large population-based sample of adult twins from Finland (Milan et al, 1998). They write that we concluded that genetic factors are not necessary to explain the distribution of BCC in twins, and raise a number of issues that they believe we should have addressed.

It should first be pointed out that our conclusion was (as stated in the last sentence of the abstract) that the results confirm the major role of environmental factors, which was based on our results from various genetic models shown in Table 4. In the Introduction, we state that genetic disorders are known to be associated with the development of BCC; in the Discussion, we suggest that these disorders do not appear to be of major importance at the population level.

The decision to emphasise the best-fitting model, CE, with shared (C) and unshared (E) environmental effects, derives from the logic of model-fitting, which is to seek a model which accounts for the observed data in the most parsimonious fashion, as advocated by the standard text on twin analyses (Neale and Cardon, 1992). Such a model is more easily falsifiable in a subsequent study than a more complex model, and we look forward to other analyses of BCC from large, population-based twin or family data sets. The more complex ACE-model did indeed contain the point estimate of zero for both additive genetic effects (A) and shared environmental effects (C), but the pure environmental model, E-model, could be rejected. Nonetheless, in the ACE model, the point estimate for the additive genetic component was 7.7%, leaving over 90% of the inter-individual variability in the liability to BCC to be attributed to environmental effects. The remaining AE model, which Dr Bataille appears to be advocating, had a poorer fit than the CE or ACE models.

Precisely because of the power issue (only four MZ and seven DZ concordant pairs), we could not account for age effects in genetic modelling. It certainly would be desirable to have more twin pairs for such an analysis, but most twin study samples in the world are considerably smaller than ours.

The mean age of diagnosis of the twins in the concordant pairs was 64.1 years (range 38–82 years, both extremes being MZ male twins, four out of 22 twins being diagnosed prior to age 60 years). The twins from concordant pairs were not markedly younger than were the other BCC patients on average. Failure to account for age in genetic modelling thus appears to be an unlikely explanation for not observing genetic effects.

Three-quarters (73%) of all BCCs registered with the Finnish Cancer Registry between 1953 and 1995 were located in the head and neck (unpublished data), compared with 68% of the twins from the concordant pairs of the present study. One MZ pair (ages at diagnosis 38 years and 43 years) and one DZ pair (60 and 61 years) were concordant for having a trunk location.

The identification of the role of the patched gene in the pathogenesis of BCC is a very important observation, which we also indicated (with three references) in our Discussion. However, the basal cell naevus syndrome is a rare disease, and accounts for only a very minor fraction of all BCCs in the population. The role of germline mutations in the patched gene in sporadic BCC cases should be assessed by the careful study of an unselected BCC

 © 2000 Cancer Research Campaign
